# LASSBio-1986 as a Multifunctional Antidiabetic Lead: SGLT1/2 Docking, Redox–Inflammatory Modulation and Metabolic Benefits in C57BL/6 Mice

**DOI:** 10.3390/ijms27020829

**Published:** 2026-01-14

**Authors:** Landerson Lopes Pereira, Raimundo Rigoberto B. Xavier Filho, Gabriela Araújo Freire, Caio Bruno Rodrigues Martins, Maurício Gabriel Barros Perote, Cibelly Loryn Martins Campos, Manuel Carlos Serrazul Monteiro, Isabelle de Fátima Vieira Camelo Maia, Renata Barbosa Lacerda, Luis Gabriel Valdivieso Gelves, Damião Sampaio de Sousa, Régia Karen Barbosa De Souza, Paulo Iury Gomes Nunes, Tiago Lima Sampaio, Gisele Silvestre Silva, Deysi Viviana Tenazoa Wong, Lidia Moreira Lima, Walter José Peláez, Márcia Machado Marinho, Hélcio Silva dos Santos, Jane Eire Silva Alencar de Menezes, Emmanuel Silva Marinho, Kirley Marques Canuto, Pedro Filho Noronha Souza, Francimauro Sousa Morais, Nylane Maria Nunes de Alencar, Marisa Jadna Silva Frederico

**Affiliations:** 1 Laboratório de Bioquímica e Farmacologia (LFB), Departamento de Farmacologia e Fisiologia, Núcleo de Pesquisa e Desenvolvimento de Medicamentos (NPDM), Faculdade de Medicina, Universidade Federal do Ceará, Rua Coronel Nunes de Melo, 1000-Rodolfo Teófilo, Fortaleza CEP 60430-275, Ceará, Brazil; landersonplopes@gmail.com (L.L.P.); gabrielaafreire@alu.ufc.br (G.A.F.); caiobruno@alu.ufc.br (C.B.R.M.); gabrielperote@alu.ufc.br (M.G.B.P.); cibellymartins95@gmail.com (C.L.M.C.); mcsam@ufc.br (M.C.S.M.); regiakarenbarbosa@hotmail.com (R.K.B.D.S.); pign23@gmail.com (P.I.G.N.); gihchemistry@gmail.com (G.S.S.); nylane@ufc.br (N.M.N.d.A.); 2 Programa de Pós-Graduação em Ciências Naturais, Universidade Estadual do Ceará, Av. Dr. Silas Munguba, 1700, Fortaleza CEP 60714-903, Ceará, Brazil; rigobertoembrapams@gmail.com (R.R.B.X.F.); damiao.sampaio@aluno.uece.br (D.S.d.S.); marinho.marcia@gmail.com (M.M.M.); helciodossantos@gmail.com (H.S.d.S.); jane.menezes@uece.br (J.E.S.A.d.M.); kirley.canuto@embrapa.br (K.M.C.); 3 Laboratório Farmacologia da Inflamação e do Câncer (LAFICA), Departamento de Farmacologia e Fisiologia, Núcleo de Pesquisa e Desenvolvimento de Medicamentos (NPDM), Faculdade de Medicina, Universidade Federal do Ceará, Rua Coronel Nunes de Melo, 1000-Rodolfo Teófilo, Fortaleza CEP 60430-275, Ceará, Brazil; isabelledefatima@gmail.com (I.d.F.V.C.M.); deysiviviana@ufc.br (D.V.T.W.); 4 Departamento de Ciências Farmacêuticas, Instituto de Ciências Biológicas e da Saúde, Universidade Federal Rural do Rio de Janeiro (UFRRJ), Seropédica CEP 23890-000, Rio de Janeiro, Brazil; renlacerda@yahoo.com.br; 5 Laboratório de Avaliação e Síntese de Substâncias Bioativas (LASSBio), Instituto de Ciências Biomédicas, Universidade Federal do Rio de Janeiro (UFRJ), Rio de Janeiro CEP 21941-590, Rio de Janeiro, Brazil; luisga011@hotmail.com (L.G.V.G.); lmlima23@gmail.com (L.M.L.); 6 Departamento de Análises Clínicas e Toxicológicas, Universidade Federal do Ceará, Fortaleza CEP 60430-160, Ceará, Brazil; tiagosampaio91@gmail.com; 7 Consejo Nacional de Investigaciones Científicas y Técnicas (CONICET), Instituto de Investigaciones em Fisicoquímica de Córdoba (INFIQC), Córdoba X5000HUA, Argentina; water.plaez@unc.edu.ar; 8 Embrapa Agroindústria Tropical, Rua Dra, Sara Mesquita, 2270-Pici, Fortaleza CEP 60020-181, Ceará, Brazil; 9 Laboratory of Pharmacogenetics, Center for Drug Research and Development (NPDM), Universidade Federal do Ceará, Fortaleza CEP 60355-636, Ceará, Brazil; pedrofilhobio@gmail.com; 10 Laboratory of Bioinformatics Applied to Human Health, Department of Pathology and Legal Medicine, Universidade Federal do Ceará, Fortaleza CEP 60355-636, Ceará, Brazil; 11 National Institute of Science and Technology in Human Pathogenic Fungi (FunVir), Ribeirão Preto CEP 14040-903, São Paulo, Brazil; 12 Instituto Federal do Amazonas-Campus Manaus Centro Av. Sete de Setembro, 1975. Centro, Manaus CEP 69020-120, Amazonas, Brazil; francimauro.morais@ifam.edu.br

**Keywords:** N-acylhydrazone, SGLT1, SGLT2, glucose metabolism, insulin resistance, GLUT-4 expression, oxidative stress, cytokines, molecular docking, type 2 diabetes mellitus

## Abstract

Type 2 diabetes mellitus (T2DM) involves chronic hyperglycemia, insulin resistance, low-grade inflammation, and oxidative stress that drive cardiometabolic and renal damage despite current therapies. Sodium–glucose cotransporter (SGLT) inhibitors have reshaped the treatment landscape, but residual risk and safety concerns highlight the need for new agents that combine glucose-lowering efficacy with redox–inflammatory modulation. LASSBio-1986 is a synthetic N-acylhydrazone (NAH) derivative designed as a gliflozin-like scaffold with the potential to interact with SGLT1/2 while also influencing oxidative and inflammatory pathways. Here, we integrated *in silico* and *in vivo* approaches to characterize LASSBio-1986 as a multifunctional antidiabetic lead in murine models of glucose dysregulation. PASS and target class prediction suggested a broad activity spectrum and highlighted transporter- and stress-related pathways. Molecular docking indicated high-affinity binding to both SGLT1 and SGLT2, with a modest energetic preference for SGLT2, and ADME/Tox predictions supported favorable oral drug-likeness. *In vivo*, intraperitoneal LASSBio-1986 improved oral glucose tolerance and reduced glycemic excursions in an acute glucose challenge model in C57BL/6 mice, while enhancing hepatic and skeletal muscle glycogen stores. In a dexamethasone-induced insulin-resistance model, LASSBio-1986 improved insulin sensitivity, favorably modulated serum lipids, attenuated thiobarbituric acid-reactive substances (TBARS), restored reduced glutathione (GSH) levels, and rebalanced pro- and anti-inflammatory cytokines in metabolic tissues, with efficacy broadly comparable to dapagliflozin. These convergent findings support LASSBio-1986 as a preclinical, multimodal lead that targets SGLT-dependent glucose handling while mitigating oxidative and inflammatory stress in models relevant to T2DM. Chronic disease models, formal toxicology, and pharmacokinetic studies, particularly with oral dosing, will be essential to define its translational potential.

## 1. Introduction

T2DM is a chronic, progressive metabolic disease characterized by hyperglycemia, insulin resistance, and β-cell dysfunction that collectively promote micro- and macrovascular damage [1,2,3]. Despite the wide availability of pharmacological classes—including metformin, sulfonylureas, thiazolidinediones, GLP-1 receptor agonists, and DPP-4 inhibitors—many patients fail to achieve durable glycemic control because of declining efficacy, hypoglycemia, weight gain, gastrointestinal intolerance, and treatment fatigue [4,5]. Despite substantial advances in lifestyle interventions and pharmacotherapy, many patients remain with suboptimal glycemic control and a high burden of cardiometabolic complications. In addition, current drugs can induce weight gain, hypoglycemia, gastrointestinal intolerance, or treatment fatigue, and do not fully address the inflammatory and oxidative components of the disease. Thus, there is a continuing need for therapeutic strategies that combine glucose-lowering efficacy with broader protection against metabolic stress pathways [4,5,6,7,8].

Sodium–glucose cotransporter 2 (SGLT2) inhibitors such as dapagliflozin and empagliflozin lower blood glucose by promoting renal glucosuria and have demonstrated cardiovascular and renal benefits in large clinical trials [9,10,11,12]. However, they also present limitations, including a risk of genital infections, volume depletion, and, in specific settings, ketoacidosis. Moreover, SGLT1, which is highly expressed in the intestine and heart, can contribute both to therapeutic effects and potential safety concerns when inhibited. The extent to which individual SGLT inhibitors modulate oxidative stress and inflammation in a tissue-selective manner is still being defined, and there is interest in new scaffolds capable of integrating transporter modulation with redox–inflammatory actions [13,14,15,16,17].

In parallel, the N-acylhydrazone (NAH) scaffold has gained increasing attention in medicinal chemistry as a privileged structure capable of engaging multiple biological targets [18,19,20,21,22,23]. NAH derivatives have been explored in several therapeutic areas, including infectious and inflammatory diseases, often displaying favorable physicochemical profiles and the ability to form hydrogen-bonding networks within enzyme or receptor cavities [18,24,25,26,27,28]. Previous work on NAH-based ligands, including compounds structurally related to LASSBio-1986, has characterized their antiparasitic activity and provided ADME predictions using tools such as SwissADME, but their contribution to SGLT1/2 modulation and antidiabetic effects remains largely unexplored [29,30,31,32].

LASSBio-1986 is a chemically synthesized NAH derivative designed as a gliflozin-like analog with the potential to engage SGLT1/2 while also influencing oxidative and inflammatory pathways. However, its antidiabetic, antioxidant, and anti-inflammatory properties have not been previously characterized in metabolic models [31,32,33].

The present study was therefore designed to comprehensively characterize LASSBio-1986 through an integrated *in silico*–*in vivo* strategy, combining PASS and docking-based target profiling, ADME/Tox prediction, and functional evaluation in an acute glucose challenge and a dexamethasone-induced insulin-resistance model in C57BL/6 mice. Rather than claiming definitive efficacy in T2DM or its complications, we position LASSBio-1986 as a preclinical multifunctional lead whose performance in these models motivates further investigation in chronic T2DM settings.

## 2. Results

### 2.1. PASS-Based Biological Activity Prediction and Target Class Profiling

PASS analysis revealed that LASSBio-1986 displays a broad predicted activity spectrum with high Pa values for antineoplastic (Pa = 0.828) and anti-inflammatory (Pa = 0.617) actions, along with relevant probabilities of antibacterial (Pa = 0.517), antifungal (Pa = 0.580) and antimycobacterial (Pa = 0.496) activities, among others (Table 1). The compound also showed a moderate probability of antidiabetic activity (Pa = 0.223; Pi = 0.099), in the PASS prediction (classified as “symptomatic antidiabetic”), suggesting potential benefits on glycemic control even though this was not the top-ranked endpoint.

**Figure 1:**
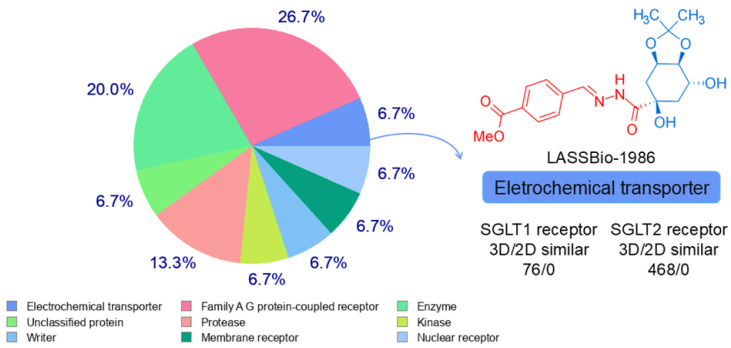
Structure-based virtual screening of target class prediction of LASSBio-1986 and similar bioactivity against SGLT1 and SGLT2 receptors in the predictive test.

Target class prediction indicated a diversified molecular interaction profile with approximately 26.7% of predicted targets belonging to GPCRs, 20.0% to enzymes, 13.3% to proteases and 6.7% to electrochemical transporters, including sodium–glucose cotransporters SGLT1 and SGLT2 (Figure 1). The convergence between transporter-related and anti-inflammatory/antioxidant predictions supports the development of a multimodal antidiabetic candidate capable of modulating both glucose handling and tissue stress responses.

### 2.2. Structural Validation and Binding Cavities of SGLT1 and SGLT2

**Figure 2:**
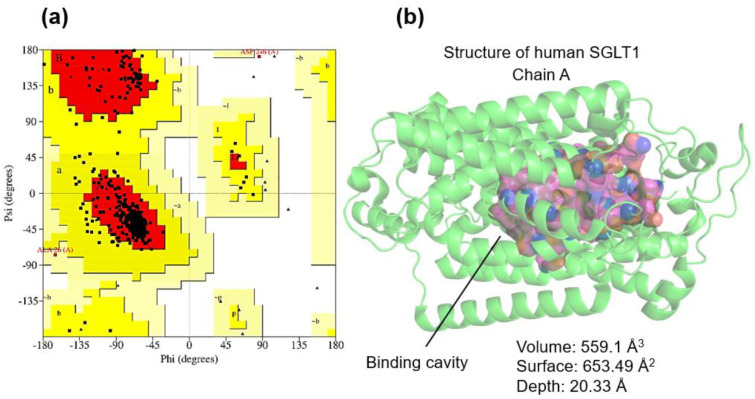
(**a**) Ramachandran plot of amino acid residues in relation to the allowed (red color), poorly allowed (yellow color), and not allowed (white color) regions. (**b**) Analysis of the SGLT1 receptor interaction cavity.

The three-dimensional structures of human SGLT1 (PDB ID: 7WMV, complexed with LX2761) and SGLT2 (PDB ID: 8HEZ, complexed with dapagliflozin) were validated by Ramachandran plots using the SAVES v6.0 platform. For SGLT1, 91.2% of residues were located in favored regions, with only Lys554 and Lys567 outside permissive zones, none of them directly involved in the catalytic site. The main helical (ψ ≈ −135°) and β-sheet (ψ ≈ 135°) distributions reflected a well-packed transmembrane architecture (Figure 2a).

Binding-cavity analysis (DoGSiteScorer, Proteins.plus) (Hamburg, Germany) identified a prominent SGLT1 pocket (volume 559.1 Å^3^, surface 653.49 Å^2^, depth 20.33 Å) comprising 35 residues, including Asn78, Leu87, Thr90, Ile98, Phe101, Ala160, Asp161, Leu274, Tyr290, Phe453, Asp454, Gln457 and Tyr526, which collectively define the glucose/inhibitor-binding region (Figure 2b).

**Figure 3:**
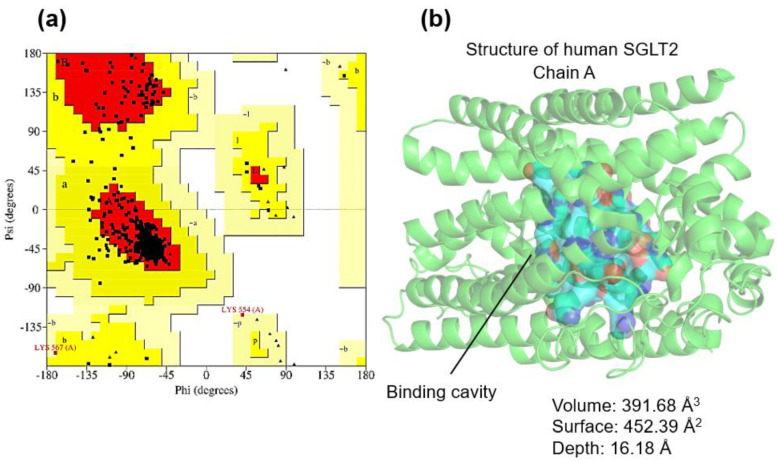
(**a**) Ramachandran plot of amino acid residues in relation to the allowed (red color), poorly allowed (yellow color) and not allowed (white color) regions. (**b**) Studies of the SGLT2 receptor interaction cavity.

For SGLT2, 90.1% of residues were found in favored regions, with minimal outliers (Asp246, Ala26) not participating in the canonical binding site (Figure 3a). The main interaction cavity displayed volume 391.68 Å^3^, surface 452.39 Å^2^ and depth 16.18 Å, with 36 key residues such as Asn75, Gly79, His80, Leu84, Thr87, Val95, Phe98, Asn101, Ala102, Ser287, Tyr290, Trp291, Lys321, Phe453, Gln457 and Tyr526 (Figure 3b). The high degree of structural similarity between SGLT1 and SGLT2 pockets is consistent with their shared substrate specificity and the clinical cross-reactivity of SGLT inhibitors.

### 2.3. Molecular Docking Against SGLT1

Docking simulations (AutoDock Vina, version 1.5.7) using a comprehensive grid encompassing the transmembrane domain revealed that LASSBio-1986 binds to the SGLT1 cavity with a predicted affinity energy (EA) of −8.2 kcal/mol, outperforming dexamethasone (−7.3 kcal/mol) and approaching the co-crystallized inhibitor LX2761 (−9.6 kcal/mol).

Root-mean-square deviation (RMSD) values for best poses remained below 2.0 Å (1.693 Å for LASSBio-1986; 1.415 Å for DEX; 1.818 Å for LX2761), indicating convergent conformational solutions and reliable binding modes (Table 2).

**Figure 4:**
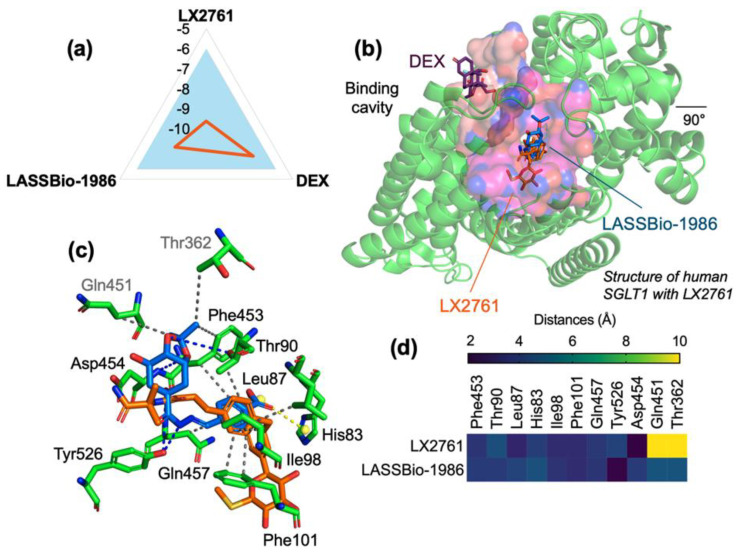
(**a**) Affinity energy graph of the SGLT1 receptor, (**b**) visualization of the binding site of the LX2761 (inhibitor—orange), DEX (commercial comparative—purple) and LASSBio-1986 (blue), (**c**) ligand-receptor interaction based on calculated distance in the amino acid residues of the SGLT1 receptor binding site and (**d**) Heatmap of interaction distances between LX2761 and LASSBio-1986 with gradient (blue—shorter distances and yellow—longer distances).

LASSBio-1986 established multiple hydrophobic contacts with Leu87, Ile98, Phe101, Thr362, Gln451 and Phe453, as well as hydrogen bonds with Thr90, Asp454 and Tyr526. In addition, a salt bridge with His83 reinforced electrostatic stabilization within the cavity. LX2761, in turn, formed an even denser network of hydrophobic and H-bond interactions with Asn78, Thr287, Leu274, Phe101, Phe453, Asp454, Gln457 and Tyr526, besides a π-cation interaction with His83, explaining its superior affinity (Figure 4).

Overall, LASSBio-1986 and LX2761 showed significant overlap in the occupied cavity and interaction pattern, suggesting that the NAH scaffold of LASSBio-1986 adequately mimics critical contacts of a clinically optimized SGLT1 inhibitor.

### 2.4. Molecular Docking Against SGLT2

In the SGLT2 model, dapagliflozin (DAP) displayed the highest affinity (−9.9 kcal/mol; RMSD 1.126 Å), consistent with its role as co-crystallized inhibitor and clinical reference. LASSBio-1986 reached −9.2 kcal/mol (RMSD 1.462 Å), again surpassing dexamethasone (−7.9 kcal/mol; RMSD 1.919 Å).

**Figure 5:**
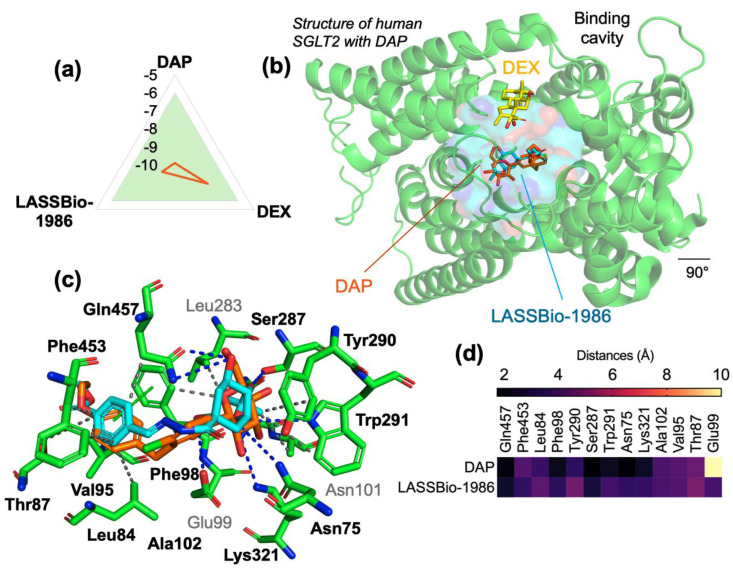
(**a**) Affinity energy graph of the SGLT2 receptor, (**b**) visualization of the binding site of the with DAP (inhibitor—orange), DEX (commercial comparative—yellow) and LASSBio-1986 (cyan), (**c**) ligand-receptor interaction based on calculated distance in the amino acid residues of the SGLT1 receptor binding site and (**d**) Hetmap of interaction distances between DAP and LASSBio-1986 with gradient (black—shorter distances and nude—longer distances).

DAP formed an extensive network of hydrophobic interactions with His80, Leu84, Val95, Phe98, Leu274, Tyr290, Phe453 and Ile456, along with multiple hydrogen bonds (Asn75, Phe98, Ala102, Ser287, Tyr290, Trp291, Lys321 and Gln457), π–π stacking with His80 and Phe98, and a halogen bond with Gly79. LASSBio-1986 recapitulated many of these interactions, engaging Leu84, Thr87, Val95, Phe98, Leu283, Tyr290, Phe453 and Gln457 through hydrophobic contacts, while forming hydrogen bonds with Asn75, Glu99, Ser287, Trp291, Lys321 and Gln457, and a π-stacking interaction with Phe98 (Table 3; Figure 5).

Both DAP and LASSBio-1986 occupied the same SGLT2 binding pocket, whereas DEX bound to an adjacent, less optimized region (Figure 5). The similarity of LASSBio-1986 to DAP in terms of interaction pattern and cavity occupancy supports SGLT2 as a primary molecular target and explains the strong antihyperglycemic effects observed *in vivo*.

### 2.5. Drug-likeness, Physicochemical and ADME/Tox Predictions

**Figure 6:**
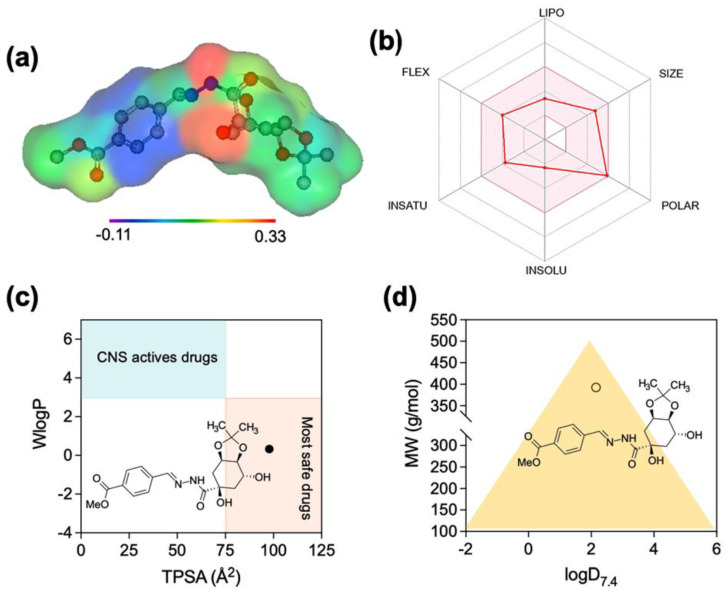
Pharmacokinetics for constituents of LASSBio-1986. (**a**) Lipophilicity distribution. (**b**) Oral bioavailability radar based on the Lipinski and Veber parameters. (**c**) Permeability in CNS and (**d**) Permeability in cell lines. The colored zone red is the suitable physicochemical space for oral bioavailability and The red line represents the physicochemical properties of the LASSBio-1986.

Computational evaluation of physicochemical properties indicated that LASSBio-1986 complies with classical drug-likeness criteria (Lipinski, Veber). The predicted MlogP (~0.20), molecular weight (~392.4 g/mol), topological polar surface area (TPSA < 140 Å^2^), number of hydrogen bond donors/acceptors, rotatable bonds, and saturation descriptors all fell within recommended intervals for orally bioavailable compounds (Table 4; Figure 6).

The estimated logD at physiological pH (≈7.4) of ~2.15 indicated moderate lipophilicity and a tendency toward a neutral, non-protonated form, favoring passive diffusion across biological membranes and systemic distribution. BOILED-Egg analysis predicted a high probability of intestinal absorption but limited penetration across the blood–brain barrier, in agreement with the WLogP and TPSA balance and the ‘non-BBB permeant’ classification in SwissADME. SwissADME-based CYP450 profiling suggested limited risk of strong inhibitory effects on major isoforms (CYP1A2, 2C19, 2C9, 2D6, 3A4), although low-to-moderate interactions could not be excluded and warrant experimental confirmation.

ProTox-3.0 predictions indicated an acceptable acute toxicity window (LD_50_ compatible with drug-like compounds) and low-to-moderate risks for immunotoxicity, mutagenicity, hepatotoxicity, cytotoxicity, and carcinogenicity, with probability values and structural alerts compatible with further preclinical development (Table 5 and Table 6).

### 2.6. LASSBio-1986 Improves Glucose Tolerance in C57BL/6 Mice

**Figure 7:**
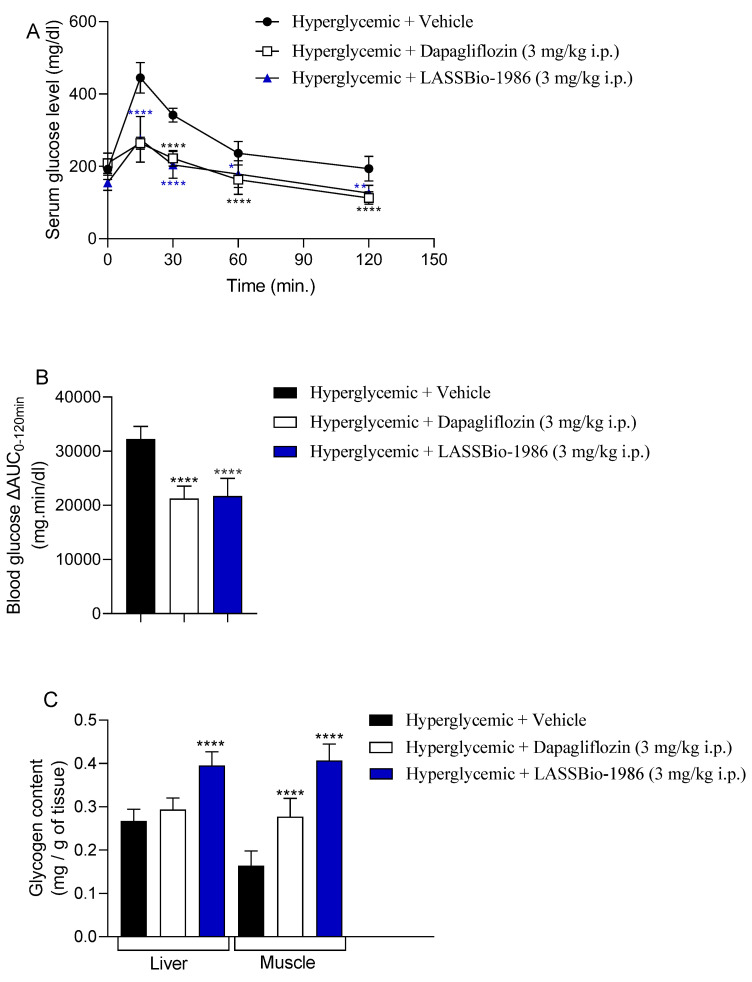
(**A**) Dose–response curve of LASSBio-1986 and Dapagliflozin 3 mg/kg i.p. on GTT and (**B**) The AUC_0_–_120_ min for the GTT was calculated from the mean glucose values of seven mice per group at each time point. (**C**) Glycogen content of mice that received the compound LASSBio-1986 administered via i.p. on GTT in skeletal muscle and liver. Values are expressed as mean ± SD; *n* = 7. * *p* ≤ 0.05, ** *p* ≤ 0.01, **** *p* ≤ 0.0001, compared to the hyperglycemic group (2 g/kg).

In a glucose tolerance test (GTT) performed in fasted C57BL/6 mice, LASSBio-1986 (3 mg/kg, i.p.) reduced serum glucose levels at 15, 30, 60, and 120 min following a 2 g/kg glucose challenge to an extent comparable to dapagliflozin at the same dose. Relative to the hyperglycemic control group, LASSBio-1986 reduced glycemia by approximately 38%, 40%, 24% and 34% at these respective time points (Figure 7A; Table 7).

The area under the curve (AUC) was significantly decreased by both LASSBio-1986 and dapagliflozin, indicating improved glucose tolerance and attenuation of post-load glycaemic peaks (Figure 7B). Importantly, the magnitude and kinetics of the antihyperglycaemic effect of LASSBio-1986 were similar to those of dapagliflozin, reinforcing its functional relevance as an SGLT-modulating scaffold.

### 2.7. Modulation of Glycogen Stores, Oxidative Stress and Inflammatory Markers

**Figure 8:**
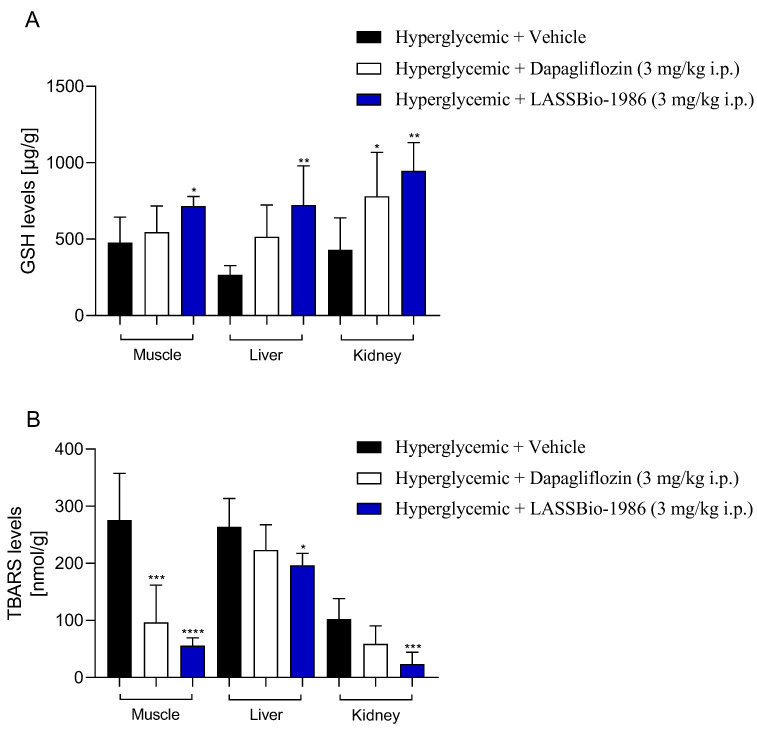
Effect of LASSBio-1986 (3 mg/kg i.p.) and Dapagliflozin (3 mg/kg i.p.) on the levels of oxidative stress biomarkers in hepatic, renal, and muscular tissues from C57B/6 mice. (**A**) GSH concentration. (**B**) TBARS content. Values are expressed as mean ± SD; n = 7. * *p* ≤ 0.05, ** *p* ≤ 0.01, *** *p* ≤ 0.001, **** *p* ≤ 0.0001, compared to the hyperglycemic group (2 g/kg).

Daily administration of LASSBio-1986 (3 mg/kg) to insulin-resistant mice increased hepatic and skeletal muscle glycogen content relative to dexamethasone-treated controls, indicating improved glucose uptake and storage in peripheral tissues (Figure 7C). Parallel increases in reduced glutathione (GSH) levels were observed in liver, muscle and kidneys, while thiobarbituric acid reactive substances (TBARSs) were significantly reduced, demonstrating a robust antioxidant effect and attenuation of lipid peroxidation (Figure 8).

**Figure 9:**
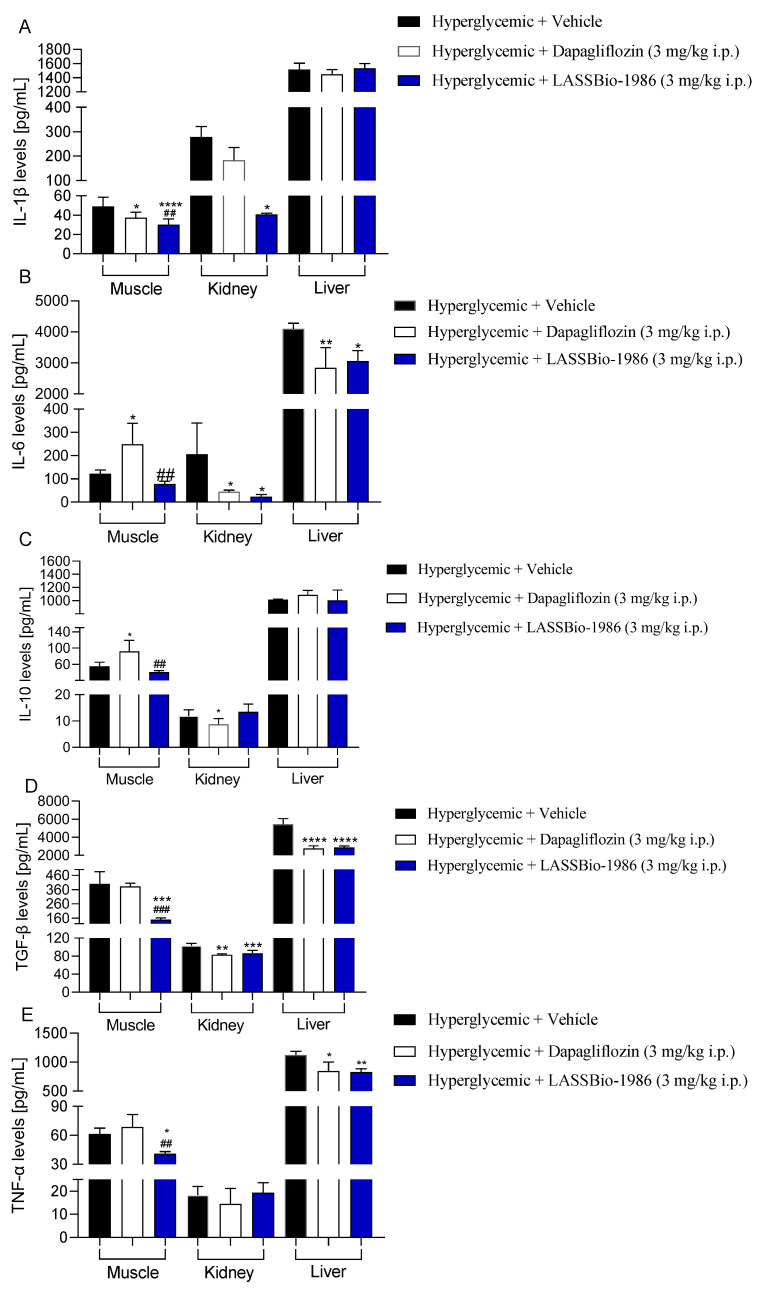
Effect of LASSBio-1986 (3 mg/kg i.p.) and Dapagliflozin (3 mg/kg i.p.) on (**A**) IL-1β, (**B**) IL-6, (**C**) IL-10, (**D**) TGF-β, (**E**) TNF-α levels in muscle, kidney and liver tissues of C57Bl/6 mice. Values are expressed as mean ± SD; n = 7. * *p* ≤ 0.05, ** *p* ≤ 0.01, *** *p* ≤ 0.001, **** *p* ≤ 0.0001, compared to the hyperglycemic group (2 g/kg) and ^##^ *p* ≤ 0.01, ^###^ *p* ≤ 0.001, compared to the Dapagliflozin group (3 mg/kg).

In the analysis of tissue cytokines (Figure 9), we characterized inflammatory and profibrogenic mediators in skeletal muscle, kidney and liver from diabetic mice. Overall, LASSBio-1986 (3 mg/kg, i.p.) markedly reduced IL-1β, IL-6, TGF-β and TNF-α while maintaining IL-10 levels within a balanced range across these target tissues, yielding cytokine profiles comparable to those observed with dapagliflozin (3 mg/kg, i.p.). These findings are consistent with the experimental model employed and with the predicted anti-inflammatory and cytoprotective spectrum of LASSBio-1986, and they are in line with previous reports of NAH derivatives exhibiting combined antioxidant and anti-inflammatory actions.

### 2.8. Effects on Insulin Sensitivity and GLUT-4 Expression

**Figure 10:**
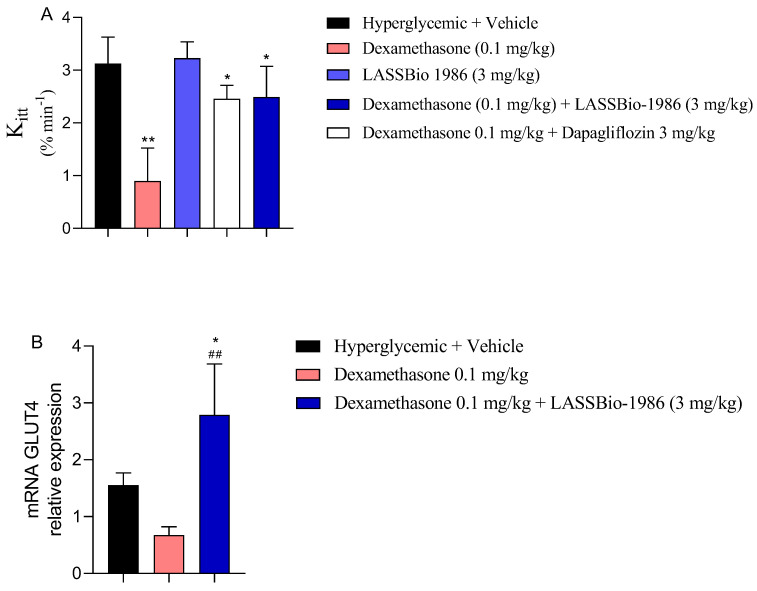
Effect of LASSBio-1986 treatment on (**A**) insulin sensitivity in insulin-resistant animals and (**B**) GLUT4 expression in the skeletal muscle tissue. Values are expressed as mean ± SD; n = 7. * *p* ≤ 0.05, ** *p* ≤ 0.01, compared to the hyperglycemic group (2 g/kg) and ^##^ *p* ≤ 0.01, compared to the Dexamethasone group (0.1 mg/kg).

In the dexamethasone-induced insulin resistance model, LASSBio-1986 restored insulin sensitivity, as evidenced by improved insulin tolerance test responses and partial normalization of glycemic excursions following exogenous insulin administration (Figure 10A). Moreover, LASSBio-1986 upregulated GLUT-4 mRNA expression in skeletal muscle (Figure 10B), counteracting the typical glucocorticoid-mediated downregulation of glucose transporters.

Given that GLUT-4-mediated skeletal muscle glucose uptake is a rate-limiting step in whole-body glucose homeostasis, preservation and enhancement of GLUT-4 expression represent a central mechanism by which LASSBio-1986 improves glycemic control.

### 2.9. Improvement of Lipid Profile

LASSBio-1986 favorably modulated serum lipids in dexamethasone-treated mice. Compared with the hyperglycemic group, LASSBio-1986 reduced total cholesterol and triglycerides and tended to increase HDL cholesterol, with a profile comparable to dapagliflozin (Table 8). Notably, when combined with dexamethasone, LASSBio-1986 significantly attenuated dexamethasone-induced dyslipidemia, reducing total cholesterol and triglycerides while normalizing HDL levels.

These pleiotropic effects on lipid metabolism complement the antihyperglycemic, antioxidant, and anti-inflammatory actions, indicating a broader cardiometabolic protective profile.

## 3. Discussion

This study combined *in silico* and *in vivo* approaches to characterize LASSBio-1986, a multifunctional N-acylhydrazone (NAH) derivative, in an acute glucose overload model and in a dexamethasone-induced insulin resistance model in C57BL/6 mice. In these settings, LASSBio-1986 consistently improved oral glucose tolerance, reduced glycemic excursions, increased hepatic and skeletal muscle glycogen stores, and attenuated oxidative and inflammatory markers, with efficacy comparable to dapagliflozin. These findings are particularly relevant in the context of T2DM, in which current therapeutic tools—despite the growing arsenal of agents such as metformin, sulfonylureas, GLP-1 receptor agonists and SGLT2 inhibitors—still leave a substantial proportion of patients with suboptimal control and high residual cardiometabolic risk [1,2,3,4,5,8,9,10]. By integrating glycemic control with redox and inflammatory modulation, LASSBio-1986 aligns with the emerging paradigm that effective T2DM management must simultaneously address glucose, inflammation and oxidative stress, rather than glycemia alone [4,5,8,9,10,11,12].

PASS and SwissTargetPrediction analyses indicated a broad putative activity spectrum for LASSBio-1986 and highlighted transporters and stress-response pathways as relevant target classes. Interestingly, the probabilistic PASS output did not rank antidiabetic activity among the main predicted endpoints, even though the compound exhibited robust *in vivo* effects on glucose tolerance and insulin sensitivity. This apparent discrepancy underscores the limitations of purely statistical/computational prediction models, which are trained on historical chemotypes and may underrepresent less common designs, such as NAH derivatives, or chemotypes originally developed for non-metabolic indications [18,19,23,25]. Similar gaps between *in silico* predictions and experimental performance have been described for other NAH-based ligands that later proved to exert multitarget actions on enzymes, receptors and inflammatory mediators [18,19,20,21,24,25,26,27,28]. Our data reinforce the view that *in silico* tools are most valuable when integrated with experimental validation and structure-based docking—rather than used as standalone filters—to reveal non-obvious pharmacological profiles and opportunities for repurposing molecular designs [18,19,20,21,25,26,27,28,30,34,35,36,37,38].

The predicted ADME and toxicity profile of LASSBio-1986 supports its development potential, with favorable lipophilicity, molecular size, polarity and gastrointestinal absorption parameters according to well-established *in silico* conceptual frameworks, such as ESOL solubility estimation, Lipinski’s criteria and the bioavailability score [34,36,37]. These properties are broadly compatible with the oral drug-likeness observed for many clinically used SGLT2 inhibitors and second-generation NAH derivatives [9,10,11,18,19,25,29]. It is important to emphasize, however, that all efficacy experiments in the present work were carried out via intraperitoneal administration. Thus, although the ADME data provide a rational basis for pursuing oral dosing, they do not substitute for experimental pharmacokinetic and bioavailability evaluations. Dedicated PK/PD studies—including oral administration, exposure profiling in plasma and tissues, and exposure–response modeling—will be essential to define the attainable therapeutic window and to confirm whether the predicted oral drug-likeness in fact translates into adequate systemic and tissue exposure in both healthy and diabetic conditions [10,29,30,31,34,35,36,37,38].

From a functional perspective, LASSBio-1986 improved oral glucose tolerance and reduced fasting and post-challenge glycemia in the acute glucose overload model, while increasing glycogen content in liver and skeletal muscle. In the dexamethasone protocol, LASSBio-1986 improved insulin sensitivity, attenuated dyslipidemia, reduced TBARS levels and restored GSH in metabolically active tissues. These integrated effects on glucose handling, redox balance and lipid homeostasis are consistent with a multimodal profile in which SGLT-related mechanisms intersect with broader metabolic stress pathways. The magnitude and coherence of these effects are comparable to those described for SGLT2 inhibitors and other insulin-sensitizing agents that simultaneously modulate glycemia, lipid profile and markers of oxidative stress, although our models are shorter in duration and less complex than chronic T2DM models [4,5,9,10,11,12,29,30,31,39]. Even so, the present design does not allow us to clearly distinguish whether the antioxidant and anti-inflammatory effects are direct pharmacological actions or secondary consequences of better glycemic control and reduced glucotoxicity. Future studies should address this question using cell-free and cellular systems to investigate radical-scavenging capacity and signaling nodes such as NF-κB and NLRP3, ideally in parallel with direct transporter and kinase-signaling assays [18,22,24,25,26,27,28].

Oxidative stress and low-grade inflammation are recognized hallmarks of T2DM and its complications, contributing to endothelial dysfunction, tissue fibrosis and organ failure [4,5,9,10,11,12]. In our models, LASSBio-1986 reduced lipid peroxidation and rebalanced cytokine networks in skeletal muscle, liver and kidney, including modulation of IL-1β, IL-6, TNF-α, IL-10 and TGF-β1. The use of validated colorimetric and spectrophotometric methods for glycogen, GSH and TBARS measurements supports the robustness of these findings at the biochemical level [40,41,42,43,44]. The attenuation of renal TGF-β1 is particularly relevant given its central role in profibrotic signaling and progression to nephropathy. Although our experiments were not designed to model established diabetic nephropathy or cardiomyopathy, the pattern of redox and cytokine modulation observed with LASSBio-1986 resembles that reported for other phenolic-rich or multitarget antidiabetic interventions that combine glucose lowering with direct antioxidant and anti-inflammatory actions [24,25,28,39,45]. Therefore, the current data indicate that LASSBio-1986 can influence pathways relevant to diabetic complications, but they do not constitute direct evidence of protection in chronic complication models. Long-term studies in validated models of nephropathy, cardiomyopathy and vascular disease will be needed in the future to address this point.

From a chemical biology standpoint, the profile of LASSBio-1986 is aligned with the growing body of evidence that NAH scaffolds can be tuned to act not only on classical inflammatory targets (such as COX, LOX, iNOS and cytokine networks) but also on metabolic endpoints, including obesity, lipid metabolism and incretin signaling [18,19,20,21,22,24,25,26,27,28]. NAH derivatives with anti-obesity activity and improved safety profiles, dual COX/LOX-modulating NAHs and β-amino-NAHs acting as DPP-4 inhibitors illustrate the versatility of this scaffold in addressing complex, multifactorial diseases [25,26,27]. Our data extend this concept to the modulation of glucose transport and redox-inflammatory axes in a context of glucocorticoid-induced metabolic stress. In this sense, LASSBio-1986 can be viewed as part of a broader class of synthetic and semi-synthetic multitarget agents that integrate insulin-independent glucose lowering with organ-protective mechanisms, analogous to what has been described for certain phenolic-rich plant extracts and small molecules such as ursolic acid [39,46].

Glucocorticoid-induced insulin resistance is a clinically relevant paradigm that recapitulates hyperglycemia, dyslipidemia and changes in glucose transporters, including GLUT-4, under conditions of increased steroid exposur—a scenario observed in patients on chronic glucocorticoid therapy. In this context, LASSBio-1986 increased insulin sensitivity, enhanced GLUT-4 expression in skeletal muscle and attenuated dexamethasone-induced dyslipidemia, again underscoring the advantage of a multimodal profile. Comparable improvements in insulin tolerance tests and GLUT-4-mediated glucose uptake have been reported with other insulin-sensitizing interventions, including phenolic-rich extracts and natural products with insulin-mimetic properties, which often act through convergent kinase- and calcium-dependent signaling pathways [39,45,46,47]. Our findings suggest that LASSBio-1986 may act at the intersection between SGLT-mediated glucose reuptake and insulin-dependent glucose uptake in peripheral tissues, positioning NAH-based ligands as promising tools to address steroid-induced metabolic disturbances, for which therapeutic options remain limited.

Docking simulations indicated that LASSBio-1986 exhibits high predicted affinity for both SGLT1 and SGLT2, with a modest energetic preference for SGLT2 and a binding mode that recapitulates key pharmacophoric interactions of clinically used inhibitors [14,29,30,32,33]. This dual profile is mechanistically attractive, since intestinal SGLT1 and renal SGLT2 act in a coordinated manner to regulate systemic glucose homeostasis [9,10,33]. At the same time, it raises important safety considerations, particularly regarding intestinal glucose absorption and potential cardiac effects associated with SGLT1 inhibition [11,13,14,15,16,17,29,30,31]. In our short-term experiments, LASSBio-1986 was well tolerated, with no clear signs of gastrointestinal discomfort or acute toxicity at the tested doses, and with no evidence of hypoglycemia or dehydration. However, experience with clinically used SGLT2 inhibitors shows that rare but serious adverse events—including genitourinary infections, euglycemic ketoacidosis and volume depletion—may become apparent only under chronic exposure and in vulnerable populations [10,13,14,15,16,17,29,30,31]. More detailed safety-pharmacology studies—including cardiac evaluations, assessment of gastrointestinal function and long-term monitoring in models with comorbidities—will therefore be needed before the risk–benefit balance of dual SGLT1/2 modulation can be established.

This study has some aspects that warrant consideration. First, we chose an acute glucose overload model and a short-term dexamethasone exposure protocol, which represent early investigative steps and do not fully reproduce the complexity of chronic T2DM models. In addition, formal toxicology and comprehensive PK studies have not yet been conducted, which is expected at this exploratory stage of development. Likewise, the effects on oxidative stress and inflammation were evaluated at a global tissue level, without a more refined dissection of upstream signaling cascades or cell type–specific responses, and we did not include, at this point, dedicated in vitro functional assays to comparatively quantify SGLT1 versus SGLT2 inhibition or to map potential off-target interactions. Even so, despite these limitations inherent to an initial preclinical study, the integrated *in silico*–*in vivo* body of evidence positions LASSBio-1986 as a promising candidate that clearly justifies further investigation. Future studies should encompass oral administration, detailed PK/PD characterization, chronic T2DM and complication models, direct functional assays on transporters and combination regimens with established antidiabetic drugs, in order to more precisely define the role of LASSBio-1986 in the evolving therapeutic landscape shaped by SGLT2 inhibitors and other multitarget agents [4,5,9,10,11,12,19,20,21,22,25,26,27,28,29,30,31].

By combining the NAH scaffold with a gliflozin-like topology, LASSBio-1986 emerges as a next-generation antidiabetic candidate. Its integrated *in silico*–*in vivo* profile suggests that it may complement or potentially enhance the benefits of existing SGLT2 inhibitors, offering broader metabolic and organ protection in T2DM [9,10,11,12,18,19,20,21,22,25,26,27,28,29,30,31]. Still, further optimization, mechanistic exploration and chronic *in vivo* evaluation are needed to determine its potential for clinical application and to establish whether dual SGLT1/2-modulating NAH derivatives can represent a viable, safe and effective strategy within the expanding spectrum of precision therapies for metabolic diseases.

## 4. Materials and Methods

### 4.1. Synthesis of LASSBio-1986

The reactions were monitored by thin layer chromatography (TLC) on 0.2 mm thick Kieselgel 60 (F254, Merck KGaA, Darmstadt, Germany). Purification by column adsorption chromatography was carried out using 230–400 mesh silica gel (60 F254, Merck KGaA, Darmstadt, Germany) as the stationary phase and gradient mixtures of n-hexane:ethyl acetate or dichloromethane:methanol as the mobile phase. Melting points were determined on a Quimis model Q340.23 apparatus and on a Shimadzu DSC-60 differential scanning calorimetry apparatus containing an FC-60 flow controller, TA 60WS integrator and TA 60 version 2.0 software calibrated with Indium, reference standard (156.6 °C) and transition energy of 28.45 J/g (LASSBio/UFRJ).

The nuclear magnetic resonance analyses were carried out at the Natural Products Research Institute (IPPN) in the Multiuser NMR Analysis Laboratory (LAMAR) and at the Chemistry Institute in the Solution Nuclear Magnetic Resonance Laboratory (LABRMN-2), both at the Federal University of Rio de Janeiro (UFRJ), in Bruker Avance III 500 MHz, 11.75 T, ultra-shielded, 54 mm cavity apparatus equipped with three Radio Frequency channels operating in the 200–600 MHz range, at powers of 100 W for observing the nuclei (500.13 for 1H NMR and 125.03 MHz for ^13^C NMR (IQ-UFRJ), or Varian 400-MR (NPPN-UFRJ), operating at 400, 300 and 75 MHz, respectively. The samples were dissolved in DMSO-d_6_ and placed in 5 mm diameter tubes. The chemical shifts (δ) were expressed in parts per million (ppm) from the internal standard tetramethyl silane (TMS), and the coupling constants (J) were given in Hertz (Hz). The peak areas were obtained by electronic integration and their multiplicities were described as follows: s-simplet/d-duplet/t-triplet/q-quadruplet/m-multiplet/dd-doublet. The assignments of the ^1^H and ^13^C NMR spectra peaks for the compounds obtained were based on the two-dimensional spectra (COSY, HMBC, HSQC) and DEPT-135. MestreNova^®^ software version 6.1.0-6224 (Mestrelab Research L.S^®^, San Diego, CA, USA) was used to process the spectra and obtain the chemical shift values (δ, ppm) [32].

The purity of the final products was determined using Shimadzu High Performance Liquid Chromatography—LC20AD with photo diode array detection (PDA), Kromasil 100-5-C18 column (4.6 mm × 250 mm), with a constant flow rate of 1 mL/min. The automatic injector was programmed so that the volume of sample injected per analysis corresponded to 20 µL, using acetonitrile and water 60:40 *v*/*v* as the mobile phase mixture, without pH correction. The solvents used for the analyses were of HPLC purity. High-resolution mass spectra were obtained using a hybrid quadrupole time-of-flight mass spectrometer with high mass resolution and accuracy (Xevo G2 QTof, Waters Corporation, Milford, MA, USA) with an electrospray ionization source, using samples dissolved in methanol at a concentration of 5 µg/mL and acetonitrile/water with 0.1% TFA, 1:1 in positive mode.

**Figure 11:**
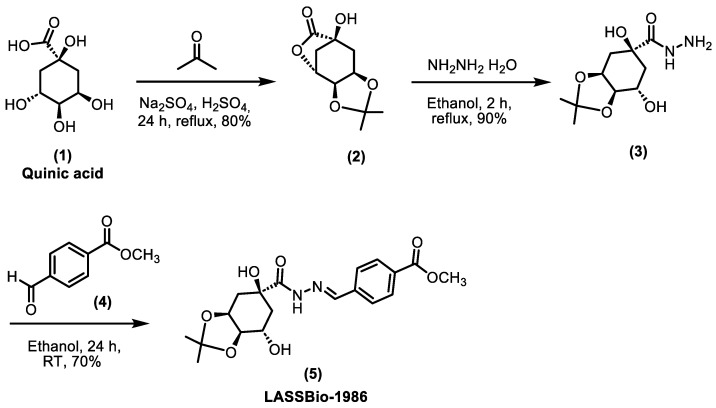
Synthetic route employed for the synthesis of the N-acylhydrazone LASSBio-1986 [32]. [(1) Quinic acid; (2) lactone–ketal intermediate; (3) hydrazide intermediate; (4) methyl 4-formylbenzoate].

The LASSBio-1986 [methyl 4-((E)-(2-((3aR,5R,7R,7aS)-5,7-dihydroxy-2,2-dimethylhexahydrobenzo[d][1,3]dioxole-5-carbonyl)hydrazineylidene)methyl)benzoate] was synthesized in three steps, as illustrated in Figure 11 [32]. The synthesis started from quinic acid (1), which was reacted with acetone under acidic conditions to protect the two cis-hydroxyl groups and form the corresponding lactone, yielding the lactone–ketal intermediate (2) with an 80% yield. This intermediate was then subjected to hydrazinolysis in the presence of one equivalent of hydrazine hydrate in ethanol under reflux, promoting ring opening of the lactone and formation of the key hydrazide intermediate (3) with a 90% yield. Due to the high polarity of the formed hydrazide, the reaction work-up involved only solvent (ethanol) evaporation under reduced pressure, without the addition of water. The N-acylhydrazone derivative LASSBio-1986 (5) was then obtained in good yields (70–72%) by condensation of hydrazide (3) with the selected aromatic aldehyde, methyl 4-formylbenzoate (4), in ethanol, without acid catalysis, to prevent possible hydrolysis of the ketal protecting group. Solid, 70% yield, HPLC 97%; ^1^H NMR (400 MHz, DMSO-d_6_) (δ ppm): 11.41 (s, 1H), 8.50 (s, 1H), 7.99 (d, 2H), 7.77 (d, 2H), 5.57 (s, 1H), 4.95 (d, 1H), 4.33 (d, 1H), 3.85 (m, 5H), 2.15 (dd, 1H), 1.85 (dd, 1H), 1.75 (dd, 1H), 1.67 (m, 1H), 1.39 (s, 3H), 1.25 (s, 3H); ^13^C NMR (126 MHz, DMSO-d_6_) (δ ppm): 173.15, 166.30, 147.13, 139.32, 130.12 (2C), 127.60 (2C), 107.90, 81.02 (2C), 74.26, 73.45, 67.39, 61.27, 52.71, 36.13, 28.62, 26.13. DEPT-135 (126 MHz, DMSO-d_6_) (δ ppm): 147.13, 130.13, 127.61, 81.03, 73.46, 67.39, 52.71, 40.53, 36,12, 28.62, 26.13. [M + H]^+^ *m*/*z*: 393.16583 (100.0%), other ions observed in the MS spectrum: 394.16859 (20%), 395.17110 (3.0%). Theoretical monoisotopic mass [M] *m*/*z*: 392.1584 (100.0%), with a mass error of ±0.94 ppm relative to the experimental value. Chemical Formula: C_19_H_24_N_2_O_7_.

### 4.2. In Silico Studies

#### 4.2.1. PASS-Based Activity Spectrum Prediction

The SMILES string of LASSBio-1986 was submitted to the PASS Online server (https://way2drug.com/PassOnline/ (accessed on 3 November 2025)), which calculates the probabilities of activity (Pa) and inactivity (Pi) for a wide range of biological endpoints. Interpretation followed conventional thresholds: Pa > 0.7 (high probability of experimentally detectable activity), 0.5 ≤ Pa ≤ 0.7 (moderate probability) and Pa < 0.5 (low probability).

#### 4.2.2. Structural Validation and Binding Cavity Prediction

The 3D structures of human SGLT1 (PDB ID: 7WMV) and SGLT2 (PDB ID: 8HEZ) were retrieved from the RCSB Protein Data Bank (https://www.rcsb.org/ (accessed on 3 November 2025)). Ramachandran plots were generated via the SAVES v6.0 server, integrating PROCHECK to assess φ and ψ dihedral angles and classify residues into favored, additionally allowed and disallowed regions.

Potential ligand-binding cavities were identified with the DoGSiteScorer module (Proteins.plus, https://proteins.plus/ (accessed on 3 November 2025)), which detects pockets based on grid-based geometry and physicochemical descriptors (volume, surface, depth, hydrophobicity and druggability score).

#### 4.2.3. Ligand Preparation and Molecular Docking

Two-dimensional molecular structures of dexamethasone (DEX), dapagliflozin (DAP) and LASSBio-1986 were obtained from PubChem (https://pubchem.ncbi.nlm.nih.gov/ (accessed on 3 November 2025)) using their SMILES representations. SMILES strings were imported into MarvinSketch (ChemAxon, academic license), and geometries were energy-minimized using the MMFF94 force field to generate low-energy conformers.

Protein preparation (removal of co-crystallized ligands LX2761 and DAP, ions and water molecules; addition of polar hydrogens; assignment of Gasteiger charges) was performed with UCSF Chimera version 1.19 and AutoDockTools. The docking grid encompassed the full transmembrane domain of each transporter: for SGLT1, box dimensions 122 × 86 × 112 Å centered at (111.304, 113.389, 114.652 Å); for SGLT2, 78 × 94 × 112 Å centered at (71.187, 67.891, 75.774 Å).

Docking simulations were performed with AutoDock Vina using exhaustiveness 64, generating 50 independent runs per ligand and 20 poses for each run. Best poses were selected based on a tripartite criterion: RMSD ≤ 2.0 Å among clustered poses, EA ≤ −6.0 kcal/mol and favorable ligand–receptor interactions (hydrogen bonds, hydrophobic contacts, salt bridges, π–π/π-cation).

#### 4.2.4. Physicochemical, Drug-likeness and ADME/Tox Prediction

The 2D structure of LASSBio-1986 was drawn in Marvin JS (ChemAxon; https://disco.chemaxon.com/calculators/demo/playground/ (accessed on 3 November 2025)) and converted to a 3D conformer for physicochemical and drug-likeness evaluation. Key descriptors, including molecular weight, calculated logP, topological polar surface area (tPSA), number of hydrogen-bond donors/acceptors, and rotatable bonds, were obtained using SwissADME (http://www.swissadme.ch/ (accessed on 3 November 2025)). The bioavailability radar was used to visualize compliance with oral drug-likeness boundaries (LIPO, SIZE, POLAR, INSOLU, INSATU, FLEX), and Lipinski, Veber, and related rules were inspected qualitatively to support the developability of the scaffold.

The BOILED-Egg model (SwissADME) was applied to estimate passive gastrointestinal absorption and blood–brain barrier (BBB) permeation, while PreADMET and related tools were used to predict plasma protein binding, cytochrome P450 interactions, and basic toxicity parameters. For LASSBio-1986, these predictions were generated de novo in the present study, building upon but not duplicating ADME analyses previously reported for other NAH derivatives. The *in silico* profile was interpreted as supportive of further preclinical development, while recognizing that experimental pharmacokinetic and safety studies are required to validate and refine these predictions.

### 4.3. In Vivo Studies

#### 4.3.1. Animals

Male C57BL/6 mice (7 weeks old, 18–20 g) were housed in standard polycarbonate cages (maximum 5 animals per cage) under controlled environmental conditions (21 ± 2 °C, 50–60% relative humidity) with a 12 h light/dark cycle (lights on from 06:00 to 18:00, lights off from 18:00 to 06:00) and free access to standard chow and water, unless otherwise specified. Animals were allowed to acclimate to the facility for at least one week before experimental procedures. Randomization was performed by assigning animals to treatment groups using a computer-generated random sequence, and investigators responsible for outcome measurements were partially blinded to group allocation when feasible. Sample sizes were based on variance and effect sizes observed in previous studies of glucose tolerance and dexamethasone-induced insulin resistance in C57BL/6 mice, aiming to detect biologically relevant differences with adequate statistical power while minimizing animal use. All procedures were approved by the Institutional Animal Care and Use Committee (Nº 17010720-0) and complied with national guidelines and the recommendations of the National Council for the Control of Animal Experimentation (CONCEA) and the ARRIVE guidelines.

#### 4.3.2. Effects of LASSBio-1986 on Glucose Tolerance Test

Fasted mice (6 h) were randomly allocated into groups of 7 animals for the oral glucose tolerance test (OGTT). LASSBio-1986 was administered intraperitoneally at doses of 1, 3, or 10 mg/kg, and dapagliflozin (3 mg/kg, i.p.) was used as a reference drug; control groups received an equivalent volume of vehicle. Thirty minutes after drug or vehicle administration, mice were challenged with an oral glucose load (2 g/kg) by gavage. Blood samples were collected from the tail vein at 0 (immediately before glucose), 15, 30, 60 and 120 min after glucose administration, and glycemia was measured using a Bioland G-500 blood glucose meter (Shenzhen, Guangdong, China) via a tail cut (glucose oxidase method. The area under the glycemic curve (AUC) was calculated for each animal using the trapezoidal rule to provide an integrated index of glucose tolerance over the 180 min period [45].

#### 4.3.3. Glycogen Content Measurements

Glycogen was isolated from the liver and soleus muscle of the animals at 120 min in the GTT assay. The tissues were weighed, homogenized in 33% KOH, and boiled at 100 °C for 20 min, with occasional stirring. After cooling, 96% ethanol was added to the samples and heated to boiling, followed by cooling in an ice bath to aid the precipitation of glycogen. The homogenates were centrifuged at 3000 rpm for 15 min, the supernatant was discarded, and resolubilized in water. Glycogen content was determined by treatment with iodine reagent and the absorbance was measured at 460 nm [40]. The results were expressed as mg of glycogen/g of tissue.

#### 4.3.4. Determination of Reduced Glutathione (GSH) Concentration

Reduced glutathione (GSH) was quantified in liver, skeletal muscle, and kidney homogenates using a modified method based on the reaction with 5,5′-dithiobis(2-nitrobenzoic acid) (DTNB), as previously described [41]. Briefly, tissues were rapidly excised, rinsed in ice-cold saline, blotted dry, weighed, and homogenized in ice-cold phosphate buffer (150 mM) containing 0.02 M EDTA, at pH 7.4. Homogenates were centrifuged at 5.000 rpm for 15 min at 4 °C and the supernatant was mixed with DTNB reagent. Absorbance was measured at 412 nm in a microplate reader within a temperature-controlled range of 10–15 °C. Protein concentration in each sample was determined by the Bradford method, and GSH levels were expressed as nmol/mg protein.

#### 4.3.5. Determination of Thiobarbituric Acid-Reactive Substances (TBARS) Production

Thiobarbituric acid-reactive substances (TBARS) were measured as an index of lipid peroxidation in liver, skeletal muscle, and kidney homogenates using a standard colorimetric assay [41,42,43,44]. Aliquots of tissue homogenates were incubated with thiobarbituric acid (TBA) reagent under acidic conditions and heated at 95 °C for 30 min to promote the formation of the chromogenic malondialdehyde–TBA adduct. After cooling and clarification by centrifugation, absorbance was recorded at 532 nm in a microplate reader. TBARS concentrations were calculated using a standard curve generated with 1,1,3,3-tetramethoxypropane and normalized to the protein content of each sample, being expressed as nmol MDA equivalents/mg protein.

#### 4.3.6. Measurement of Cytokines (IL-1β, IL-6, IL-10, TGF-β and TNF-α) by ELISA

Concentrations of IL-1β, IL-6, IL-10, TGF-β1, and TNF-α were determined in liver, skeletal muscle, and kidney using commercially available sandwich ELISA (R&D Systems DuoSet, Minneapolis, MN, USA), following the manufacturer’s instructions. Briefly, tissue homogenates were prepared in ice-cold buffer containing protease inhibitors, clarified by centrifugation, and loaded in duplicate onto pre-coated ELISA plates. After incubation with specific detection antibodies and enzymatic substrates, absorbance was read at 450 nm (with appropriate reference wavelength) in a microplate reader. Cytokine concentrations were interpolated from standard curves generated with recombinant cytokines and normalized to the protein content of the corresponding homogenates, being expressed as pg/mg protein.

#### 4.3.7. Insulin Sensitivity Test

To evaluate the effect of LASSBio-1986 on insulin resistance, mice were divided into four groups: (1) Mice received vehicle (saline), (2) 0.1 mg/kg dexamethasone subcutaneously (s.c.), (3) LASSBio-1986 3 mg/kg i.p.; and (4) LASSBio-1986 10 mg/kg; i.p. plus dexamethasone (0.1 mg/kg s.c.). The mice were induced with daily subcutaneous injections from 8 h 30 min to 9 h 30 min a.m., for 5 consecutive days [39].

Mice that had been fasted for at least 4 h received subcutaneously injected insulin (2 U/kg body weight). Blood was collected from the tail at 0, 7, 14 and 28 min to determine glucose concentrations. This test measures insulin sensitivity using the constant disappearance of glucose calculated using the following formula: K_itt_ = 0.693 × 100/t_½_. Where t_½_ is the half-life of decay of glucose and was determined from the slope of the line obtained by linear regression of the natural logarithm of glucose versus time [47].

#### 4.3.8. Real-Time PCR

Muscle samples from mice treated or not with dexamethasone were ground and homogenized for RNA isolation, for which TRIzol™ Reagent (Invitrogen, Thermo Fisher Scientific, Carlsbad, CA, USA) using steel beads (4.5 mm) that were shaken in the TissueLyser LT (Qiagen, Hilden, Germany). We assessed the yield and quality of total RNA using the Epoch™ Microplate Spectrophotometer (BioTek Instruments, Winooski, VT, USA). RNA reverse transcription was performed using High-Capacity cDNA reverse transcription Kits and a Veriti 96-well Thermal cycler (Applied Biosystems, Thermo Fisher Scientific, MA, USA). The real-time PCR amplification was carried out using 2 μL of cDNA, specific primers for each gene, and SyBR Green reagent (Invitrogen), in a final volume of 10 μL. The 2^−(ΔΔCt)^ method was used to calculate the ΔΔCt values [48]. Real-time PCR was accomplished on a QuantStudio™ 3 equipment (Thermo Fisher Scientific, MA, USA) [49]. The Ppia gen was used as an internal control, and the primer sequences used are as follows: GLUT4: Forward: 5′-CGCGGCCTCCTATGAGATAC-3′; Reverse: 5′-CCTGAGTAGGCGCCAATGA-3′. The samples were normalized using the PPIA: forward 5′-CAGACGCCACTGTCGCTTT-3′ and reverse 5′-TGTCTTTGGAACTTTGTCTGGAA-3′ [46]. The results were obtained from the mean expression of five tissue samples analyzed independently.

### 4.4. Data and Statistical Analysis

Data are expressed as mean ± standard deviation (SD) for each experimental group. The Shapiro–Wilk test was used to assess the normality of data distributions, including qPCR-derived expression values, and homogeneity of variances was evaluated using Levene’s test. For comparisons involving a single factor (e.g., treatment groups at a single time point), one-way ANOVA followed by Tukey’s or Bonferroni’s post hoc test, as appropriate, was applied. Time-course data from the oral glucose tolerance test were analyzed using two-way repeated-measures ANOVA with factors “treatment” and “time”, followed by suitable post hoc comparisons when significant main effects or interactions were detected. When assumptions of normality or homoscedasticity were not met, non-parametric alternatives were considered as appropriate. In figures, statistical significance versus control or dexamethasone groups is indicated by symbols defined in each legend, and representative F values, degrees of freedom, and *p* values for the main ANOVA effects are reported in the Results. Statistical analyses were conducted using GraphPad Prism^®^ version 8.01 (San Diego, CA, USA), and differences were considered significant at *p* < 0.05.

## 5. Conclusions

LASSBio-1986, a NAH analog of SGLT2 inhibitors, exhibits a coherent *in silico*–*in vivo* profile consistent with a multifunctional antidiabetic lead in murine models of glucose dysregulation. In an acute oral glucose challenge, LASSBio-1986 improved glucose tolerance and increased tissue glycogen stores, whereas in a dexamethasone-induced insulin-resistance model it ameliorated insulin sensitivity, normalized lipid profile, reduced oxidative stress markers, and rebalance pro- and anti-inflammatory cytokines, in several endpoints paralleling the effects of dapagliflozin.

These convergent findings position LASSBio-1986 as a strong preclinical candidate for further evaluation as a multimodal modulator of SGLT-dependent glucose handling and metabolic stress pathways. However, its translational potential will depend on the demonstration of efficacy in chronic T2DM and complication models, the elucidation of its precise SGLT1/2 selectivity profile, comprehensive pharmacokinetic characterization (particularly with oral dosing), and formal toxicology and safety pharmacology studies, including gastrointestinal and cardiovascular assessments. Such investigations will be essential before considering exploratory clinical translation or combination strategies with established antidiabetic agents.

## Figures and Tables

**Table 1:** Predicted biological activities of LASSBio-1986 obtained using the PASS Online tool.

Property	Pa	Pi	Property	Pa	Pi
Antineoplastic	0.828	0.009	Cytoprotectant	0.445	0.079
Anti-inflammatory	0.617	0.028	Immunosuppressant	0.423	0.060
Antifungal	0.580	0.021	Antiviral	0.318	0.079
Antibacterial	0.517	0.015	Antinociceptive	0.343	0.151
Antimycobacterial	0.496	0.019	Antidiabetic symptomatic	0.223	0.099

Pa (probability of activity) indicates the likelihood that the compound exhibits the predicted biological effect, whereas Pi (probability of inactivity) reflects the likelihood of not displaying that effect.

**Table 2:** Data on ligand-receptor (L-R) interactions in the redocking process of the inhibitor LX2761, DEX comparative compound and molecular docking simulations of the LASSBio-1986, via the SGLT1 receptor.

Compd	EA (kcal/mol)	RMSD (Å)	Inter.Type	Residues/Distances (Å)
LX2761	−9.6	1.818	Hydrophobic	His83 (4.73), Leu87 (3.16), Thr90 (4.48), Ile98 (3.36), Ile98 (3.46), Phe101 (4.94), Phe101 (3.34), Phe101 (3.34), Ala160 (4.78), Leu274 (3.50), Tyr290 (4.27), Phe453 (3.65), Asp454 (4.26), Gln457 (4.04), Tyr526 (4.76)
			H-bond	Asn78 (4.00), Asn78 (2.60), Glu102 (3.21), Ala105 (4.12), Leu274 (3.96), Thr287 (1.75), Thr287 (4.11), Trp291 (2.32), Lys321 (2.80), Asp454 (2.09), Asp454 (2.80), Gln457 (3.76), Tyr526 (4.09)
			π-cátion	His83 (3.90)
DEX	−7.3	1.415	Hydrophobic	Phe155 (4.90), Ile443 (4.84), Ala447 (4.13), Tyr455 (3.65), Tyr455 (3.51), Tyr455 (4.09), Tyr455 (4.58), Ile459 (3.41), Phe504 (3.96), Phe504 (3.66), Phe504 (4.73), Phe504 (3.57)
			H-bond	Trp440 (4.43), Gln448 (3.09), Ser449 (4.21)
LASSBio-1986	−8.2	1.693	Hydrophobic	Leu87 (4.13), Ile98 (4.08), Ile98 (3.58), Phe101 (3.34), Phe101 (4.00), Thr362 (4.58), Gln451 (4.62), Phe453 (4.41), Phe453 (3.80), Phe453 (4.74), Gln457 (3.66)
			H-bond	Thr90 (3.77), Asp454 (3.86), Tyr526 (3.60), Tyr526 (2.13)
			Salt bridge	His83 (4.51)

Notes: DEX, Dexamethasone (comparative). LX2761 (inhibitor protein).

**Table 3:** Data on ligand-receptor (L-R) interactions in the redocking process of the inhibitor DAP, DEX comparative compound and molecular docking simulations of the LASSBio-1986, via the SGLT2 receptor.

Compd	EA (kcal/mol)	RMSD (Å)	Inter.Type	Residues/Distances (Å)
DAP	−9.9	1.126	Hydrophobic	His80 (4.91), Leu84 (3.75), Leu84 (3.67), Thr87 (4.66), Val95 (4.28), Phe98 (3.51), Phe98 (3.86), Val157 (4.46), Leu274 (4.18), Tyr290 (4.53), Phe453 (4.21), Ile456 (4.82), Gln457 (3.88)
			H-bond	Asn75 (1.73), Phe98 (2.29), Ala102 (4.05), Ser287 (1.74), Ser287 (1.96), Tyr290 (3.51), Tyr290 (3.43), Trp291 (2.37), Lys321 (2.43), Gln457 (1.97), Gln457 (2.41)
			π-Stacking	His80 (3.92), Phe98 (5.00)
			Halogen bond	Gly79 (3.29)
DEX	−7.9	1.919	Hydrophobic	Pro275 (4.30), Asp454 (3.58), Tyr455 (4.74), Ala458 (3.98), His525 (3.58), Tyr526 (3.85)
			H-bond	Gly272 (2.57), Ser508 (4.20)
LASSBio-1986	−9.2	1.462	Hydrophobic	Leu84 (4.43), Thr87 (4.99), Val95 (4.21), Phe98 (3.36), Asn101 (5.00), Ala102 (4.03), Leu283 (3.23), Tyr290 (4.82), Trp291 (4.75), Phe453 (3.54), Phe453 (3.81), Gln457 (3.29)
			H-bond	Asn75 (3.51), Glu99 (3.95), Glu99 (3.33), Ser287 (2.83), Trp291 (3.89), Lys321 (3.60), Gln457 (2.91), Gln457 (2.63)
			π-Stacking	Phe98 (5.33)

Notes: DAP, Dapagliflozin (inhibitor present in the protein structure); DEX, Dexamethasone (comparative).

**Table 4:** Predicted to be drug-like by the Lipinski and Veber parameters and pharmacokinetics by the ADME models of the SwissADME and preADMET web servers.

Properties	Results	Properties	Results
**ADME regression**	**Absorption**		
Physiological charge	0.00	P_caco-2_ in nm·s^−1^	10.03
logD at pH 7.4	2.15	WlogP	0.33
logS ESOL	−2.31	GI absorption	High
FCs^3^	0.53	HIA%	77.04
**Lipinski parameters**	**Distribution**		
MW in g.mol^−1^	392.40	P_-gp_ substrate	Yes
MlogP	0.20	P_-gp_ inhibitor	No
HBA	8	PPB	39.140
HBD	3	BBB permeant	No
**Veber parameters**	BB (C_brain_/C_blood_)	0.054	
Nrot	6	**Metabolism and Excretion**	
TPSA in Å^2^	97.79	CYP1A2 inhibitor	No
**Bioavailabiliy**		CYP2C19 inhibitor	No
Lipinski drug-like	Yes	CYP2C9 inhibitor	No
Veber drug-like	Yes	CYP2D6 inhibitor	No
*F*	0.55	CYP3A4 inhibitor	No

Notes: logD (distribution coefficient calculated in the Marvin JS software, version 24.1.0); logS ESOL (solubility coefficient by the ESOL method [34]); FCsp3 (Fraction of carbon sp3); MW (molecular weight); MlogP (partitioning coefficient by the Mauri and Lipinski models [35,36]); HBA (hydrogen bond acceptor count); HBD (hydrogen bond donor count); Nrot (number of rotatable bonds); TPSA (topological polar surface area); F (Fraction of compounds with bioavailability > 10% in rats by the Martin method [37]); Pcaco_-2_ (Caco_-2_ cells permeability); WlogP (partitioning coefficient by the Wildman models [38]); GI (Gastrointestinal absorption); HIA (human intestinal absorption); P-gp (P-glycoprotein); PPB (plasma protein binding); BBB (blood–brain barrier permeant); CYP (cytochrome P450 isoenzymes).

**Table 5:** Toxicological parameters predicted in the ProTox-3.0 web server.

Properties	Results	Results	
**Oral acute toxicity**		**Toxicity endpoints**	
LD50 (mg/kg)	3.000	Carcinogenicity	−(0.53)
Toxicity class	5	Immunotoxicity	+(0.96)
Prediction accuracy (%)	54.26	Mutagenicity	+(0.50)
**Organ toxicity**		Cytotoxicity	−(0.59)
Hepatotoxicity	−(0.55)	**Cardiac toxicity**	
Nephrotoxicity	+(0.56)	pAct	4.19 ± 0.55

**Table 6:** Toxicological target modulations predicted in the ProTox-3.0 web server.

Properties	Results	Results		Results	
Nuclear Receptor Signaling		Stress Response		Molecular Initiating Events	
AhR	−(0.90)	Nrf/ARE	−(0.90)	TTR	−(0.73)
AR	−(0.95)	HSE	−(0.90)	GABAR	−(0.65)
AR-LBD	−(0.94)	MMP	−(0.71)	NMDAR	−(0.94)
Aromatase	−(0.86)	p53	−(0.84)	AMPAR	−(0.95)
ER	−(0.83)	ATAD5	−(0.92)	AChE	−(0.66)
ER-LBD	−(0.93)	THRα	−(0.72)	PXR	+(0.51)
PPAR-Gamma	−(0.96)	THRβ	−(0.83)	VGSC	−(0.74)

Notes: AhR (Aryl hydrocarbon Receptor); AR (Androgen Receptor); AR-LBD (Androgen Receptor Ligand Binding Domain); ER (Estrogen Receptor Alpha); ER-LBD (Estrogen Receptor Ligand Binding Domain); PPAR-Gamma (Peroxisome Proliferator Activated Receptor Gamma); nrf2/ARE (Nuclear factor (erythroid-derived 2)-like 2/antioxidant responsive element); HSE (Heat shock factor response element); MMP (Mitochondrial Membrane Potential); p53 (Phosphoprotein (Tumor Suppressor) p53); ATAD5 (ATPase family AAA domain containing protein 5). THRα (Thyroid hormone receptor alpha); THRβ (Thyroid hormone receptor beta); TTR (Transtyretrin); NMDAR (Glutamate N-methyl-D-aspartate receptor); AMPAR (alpha-amino-3-hydroxy-5-methyl-4-isoxazolepropionate receptor); AChE (Achetylcholinesterase); PXR (Pregnane X receptor) and VGSC (Voltage-gated sodium channel).

**Table 7:** Effect of LASSBio-1986 and dapagliflozin on the glucose tolerance test in fasted C57BL/6 mice.

Group	Serum Glucose Levels (mg/dL) Time (min.)				
	0	15	30	60	120
Hyperglycemic (2 g/kg)	192.4 ± 10.82	444.7 ± 15.82	341.6 ± 7.29	236.3 ± 12.22	193.6 ± 12.87
Dapagliflozin (3 mg/kg)	208.7 ± 10.53	264.1 ± 6.33 ****	222.1 ± 8.18 ****	163.1 ± 15.35 *	112.7 ± 6.38 ****
LASSBio-1986 (1 mg/kg)	174.6 ± 5.28	349.4 ± 13.40 **	283.6 ± 10.91 **	215.9 ± 10.45	174.1 ± 9.36
LASSBio-1986 (3 mg/kg)	154.7 ± 7.94	274.9 ± 23.77 ****	204.0 ± 14.10 ****	178.7 ± 13.93 ****	126.3 ± 8.11 **
LASSBio-1986 (10 mg/kg)	161.9 ± 10.07	339.9 ± 13.29 ***	249.7 ± 8.63 ***	185.4 ± 13.17 *	179.3 ± 8.99

Values are expressed as mean ± SD; n = 7. * *p* ≤ 0.05, ** *p* ≤ 0.01, *** *p* ≤ 0.001, **** *p* ≤ 0.0001, compared to the hyperglycemic group (2 g/kg).

**Table 8:** Effect of daily treatment with LASSBio-1986 (3 mg/kg, i.p.) on the lipid profile of insulin-resistant mice.

Group	Lipid Profile (mg/dL)		
	Total Cholesterol	HDL-Cholesterol	Triglycerides
Saline	91.02 ± 15.87	56.34 ± 11.17 *	85.37 ± 14.43
Dexamethasone (0.1 mg/kg)	95.36 ± 24.25	35.52 ± 3.93	109.4 ± 44.18
Dexamethasone (0.1 mg/kg) +LASSBio-1986 (3 mg/kg)	56.14 ± 22.57 **	54.15 ± 14.35	68.65 ± 12.96 **
Dexamethasone (0.1 mg/kg) +Dapagliflozin (3 mg/kg)	64.40 ± 16.93 **	54.64 ± 15.61	78.77 ± 14.55
LASSBio-1986 (3 mg/kg)	67.70 ± 6.10	58.07 ± 8.06	78.55 ± 24.14

Values represent the mean ± SD of determinations from n = 7 animals per group. Data were analyzed by one-way ANOVA followed by Tukey’s post hoc test. * *p* < 0.05, ** *p* < 0.01, compared to the vehicle-treated control group.

## Data Availability

The original contributions presented in this study are included in the article. Further inquiries can be directed to the corresponding author.

## Abbreviations

The following abbreviations are used in this manuscript:

ADME	Absorption–distribution–metabolism–excretion
BBB	Blood–brain barrier
DAP	Dapagliflozin
DEX	Dexamethasone
GSH	Reduced glutathione
GTT	Glucose tolerance test
NAH	N-acylhydrazone
SGLT	Sodium–glucose cotransporter
TBARS	Thiobarbituric acid reactive substances
T2DM	Type 2 diabetes mellitus
